# Perpendicular Local Magnetization Under Voltage Control in Ni Films on Ferroelectric BaTiO_3_ Substrates

**DOI:** 10.1002/adma.201404799

**Published:** 2015-01-12

**Authors:** Massimo Ghidini, Francesco Maccherozzi, Xavier Moya, Lee C Phillips, Wenjing Yan, Jordane Soussi, Nicolas Métallier, Mary E Vickers, Nina -J Steinke, Rhodri Mansell, Crispin H W Barnes, Sarnjeet S Dhesi, Neil D Mathur

**Affiliations:** Department of Materials Science, University of CambridgeCambridge, CB3 0FS, UK; DiFeST, University of Parmaviale G. P. Usberti 7/A, Parma, 43124, Italy; Diamond Light SourceChilton, Didcot, Oxfordshire, OX11 0DE, UK; Cavendish Laboratory, University of CambridgeCambridge, CB3 0HE, UK; ISIS, Harwell Science and Innovation CampusDidcot, Oxfordshire, OX11 0QX, UK

**Keywords:** electrical control of magnetism, magnetoelectrics

Electrical control of magnetism has been demonstrated in multi­ferroic compounds^1^–^5^ and ferromagnetic semiconductors,^6^–^9^ but electrical switching of a substantial net magnetization at room temperature has not been demonstrated in these materials. This scientific and technological goal has instead been achieved using ferromagnetic films in which electrically driven magnetic changes arise due to strain^10^–^16^ or exchange bias^17^,^18^ from ferroic substrates, or changes of interfacial orbital occupation.^19^ However, in these works and other magnetoelectric studies, it is typically necessary to employ a variable magnetic field, either to repeatedly drive electrically nucleated magnetization reversal,^12^ or to interrogate changes of anisotropy,^16^,^19^,^20^ exchange bias,^21^ or coercivity.^12^,^14^,^22^

A variable magnetic field permits magnetoelectric phenomena to be investigated comprehensively, but electrical control of magnetization with no magnetic field represents a higher scientific goal. Purely electrical switching of in-plane (IP) magnetization has been demonstrated with thin-film ferromagnets,^11^–^13^,^23^ but electrical switching of out-of-plane (OOP) magnetization in zero applied magnetic field has only been demonstrated at a subset of small features, in high-quality epitaxial nanocomposites^24^ and polycrystalline heterostructures.^25^ Indeed, it is challenging to achieve an OOP anisotropy that is large enough to compete successfully with strong OOP demagnetizing fields, while arranging for this anisotropy to be significantly modified by an electric field. Here we create and destroy an OOP anisotropy in large contiguous areas of Ni films, by repeatedly overcoming the growth stress with electrically controlled strain from ferroelectric domain switching in BaTiO_3_ (BTO) substrates. This OOP anisotropy produces an OOP component of magnetization, which is manifested via the formation of magnetic stripe domains, and turned on and off via the applied voltage. To confirm our interpretation, we show that the same magnetic switching is achieved when quantitatively similar changes of strain are produced by thermally driving phase transitions in the BTO substrates.

Stripe domains^26^–^29^ arise in magnetic thin films when a uniaxial OOP anisotropy competes with the IP shape anisotropy, yielding a canted local magnetization whose OOP component alternates in sign along an IP direction, minimizing stray-field energy. The IP component of magnetization lies perpendicular to this IP direction, along the same direction in adjacent stripes. In polycrystalline films of negative-magnetostriction Ni, which show stripe domains above a critical thickness,^26^,^28^ the uniaxial OOP anisotropy arises due to isotropic IP tensile stress^30^ generated at grain boundaries during Volmer-Weber growth.^31^ This built-in stress does not come from the substrate, as stripe domains appear in free-standing polycrystalline films,^29^ and substrate choice does not influence stripe width (Supporting Information Note 1). Therefore stripe domains should be ubiquitous in our as-grown samples, even though our BTO substrates are twinned.

Strain from ferroelectric substrates such as BTO^10^,^12^–^15^ has been used to modify the IP magnetization of ferromagnetic films that are epitaxial,^10^,^32^ polycrystalline,^12^–^15^ and amorphous.^16^ These inverse-magnetostrictive processes are either discontinuous if thermally driven via structural phase transitions in BTO,^33^ as shown^10^,^14^ near 300 K (T ↔ O) and 200 K (O ↔ R) [T = tetragonal, O = orthorhombic, R = rhombohedral]; or discontinuous^10^ or quasi-continuous^15^ if driven by changes of voltage between film and back electrode to interconvert *c* domains (with OOP polarization) and *a* domains (whose polarization may lie along one of two perpendicular IP directions).

In our as-prepared Ni//BTO samples, stripe domains were as expected nominally ubiquitous in all films, and magnetoelectric effects were similar whether substrates were poled after (Samples 1–4) or before (Samples 5–6) film growth. We show that stripe domains are erased by cooling BTO through structural phase transitions, or by electrically moving 90° ferroelectric walls to convert *a* to *c* domains. Our high-resolution imaging of electrically driven changes reveals that ferromagnetic and ferroelectric domains are correlated, and that stripe domains undergo non-volatile repeatable erasure.

For demagnetized Ni//BTO in non-saturating magnetic fields, thermally driven structural phase transitions in the substrate yield sharp hysteretic jumps of OOP magnetization that complement expected^10^,^14^,^32^ jumps of IP magnetization (**Figure 1**a). On cooling, these jumps represent an increase of IP magnetization at the expense of OOP magnetization, consistent with two observations. First, there is a dramatic increase of easy-axis loop squareness that evidences the development of a strong IP magnetic anisotropy (Figure 1b) of biaxial character (Supporting Information Note 2). Second, the hard-axis saturation field increases to a value that corresponds closely to saturation magnetization *μ*_0_*M*_s_ = 0.54 T, and thus the demagnetizing field (inset, Figure 1b), evidencing complete suppression of weak OOP anisotropy. After returning to room temperature in non-saturating magnetic fields, IP and OOP magnetizations are slightly modified due to the stochastic nature of magnetic domain-wall depinning (Figure 1a), but easy and hard-axis loops are recovered (Figure 1b and inset). Therefore strains on cooling to R-phase BTO appear to be reversible.

**Figure 1:**
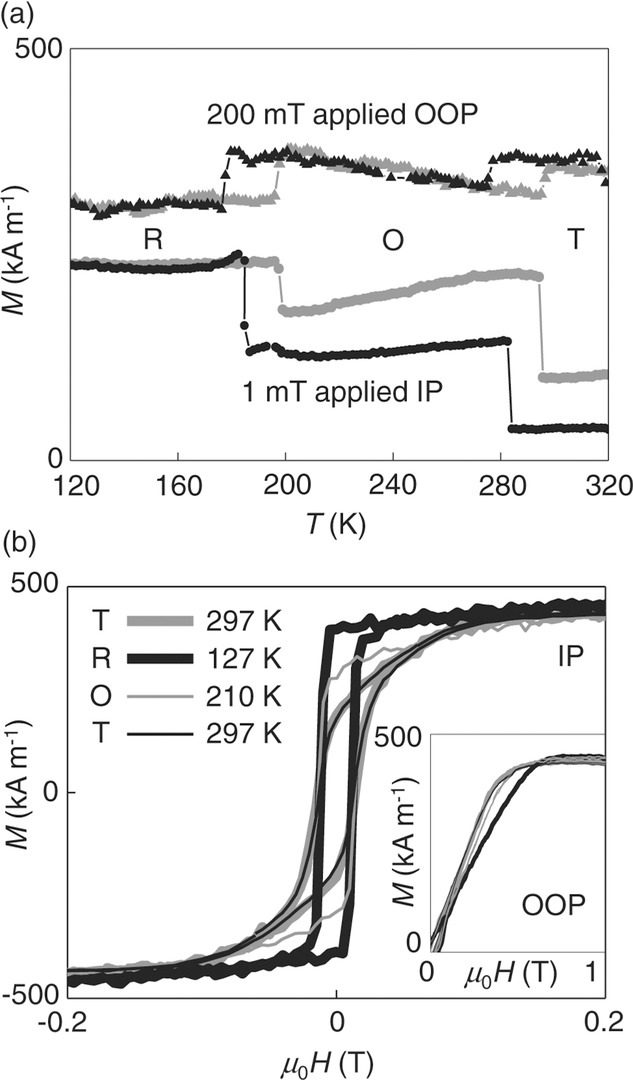
Thermal control of magnetic anisotropy in Ni//BTO. a) IP (circles) and OOP (triangles) magnetization *M* as a function of temperature *T*, measured on cooling (black) and subsequent heating (gray) in non-saturating fields. The sample was demagnetized at room temperature prior to each thermal cycle. b) IP magnetization *M* versus applied magnetic field *H* at 297 K (thick gray), 127 K (thick black), 210 K (thin gray), and 297 K again (thick black). The inset shows the corresponding OOP data. R, O, and T denote phases of BTO. Data for Sample 1.

Magnetic imaging was performed using photoemission electron microscopy (PEEM) and magnetic force microscopy (MFM). PEEM data were obtained with X-ray magnetic circular dichroism (XMCD) contrast, so images depend on the projection of local magnetization onto the grazing-incidence X-ray beam direction. Therefore the IP and OOP components of magnetization are imaged simultaneously to provide 3D information, and the alternating OOP component of magnetization in stripe domains^26^–^29^ is apparent for any IP sample orientation.

XMCD–PEEM (**Figure 2**a) revealed stripe domains with a stripe width of ≈125 nm, comparable with our 100 nm film thickness as expected.^28^ As also expected, these stripe domains were nominally ubiquitous in all of our many PEEM/MFM images of as-grown Samples 1-4, fabricated with electrically virgin BTO substrates that primarily comprised *a* domains with IP polarizations at 90° to one another (*a*_1_–*a*_2_ domains^13^). Long thin features with IP magnetizations were observed in Sample 1, and could represent an initial decoration^13^ of *c* domains with OOP polarizations (Supporting Information Note 3), but using MFM this could not be confirmed as they were not reproduced after prepoling BTO substrates to produce a significant population of *c* domains (Samples 5–6). In view of this irreproducibility, the long-thin features are considered no further, but the example in Figure 2a is helpful because the antiparallel tail-to-tail domains set the XMCD asymmetry scale for Figure 2c.

**Figure 2:**
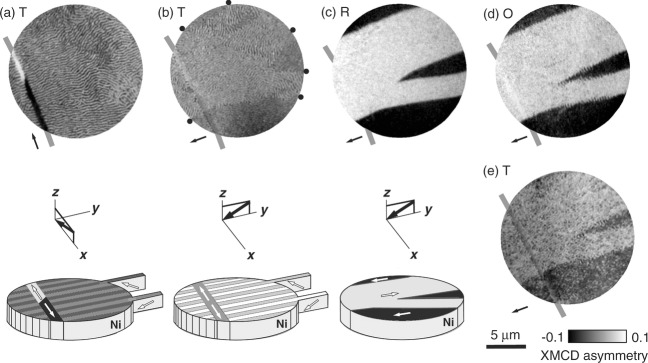
Thermal control of stripe domains. PEEM images with XMCD contrast for a single region at a) 297 K, b) after rotating the sample 90°, and then at c) 127 K, d) 210 K, and e) 297 K. Panels (a–c) include representative schematics under each image. Black arrows show the IP projection of the incident-beam direction (images), and the incident-beam direction (schematics). White arrows show magnetization directions. Schematics do not show waviness in stripe domains, or variations of IP magnetization. Black dots in (b) indicate boundaries between IP magnetic domains. Thick gray lines mark the long thin feature that is representative of this sample only (Supporting Information Note 3). Data for Sample 1.

The Figure 2a stripe orientation lies approximately perpendicular to the IP projection of the beam, and there is waviness due to inhomogeneous IP stress. Therefore the IP component of magnetization along the local stripe direction^26^–^29^ has typically no significant projection onto beam direction. On rotating the sample 90° (Figure 2b), the same field of view reveals that the locally inhomogeneous IP component of magnetization is split into a few large domains that coexist with the alternating OOP component of magnetization, which remains visible.

On cooling to the R-phase of the substrate, stripe domains are annihilated in favor of a uniform IP magnetization within each domain, now clearly visible with slightly modified boundaries (Figure 2c). This annihilation is due to suppression of the OOP anisotropy responsible for stripe domains, consistent with our macroscopic OOP data (inset, Figure 1b). The resulting IP domains are antiparallel, evidencing the development of a uniaxial IP anisotropy in our field of view, which thus contains only one of the two twin species responsible for the biaxial anisotropy (Supporting Information Note 2).

Subsequent warming to the O-phase of the substrate nucleates stripes at IP domain walls (Figure 2d). Returning to the room temperature T-phase recovers stripe domains that are less wavy than before (Figure 2e). The low-temperature excursion has rendered the IP film stress more homogenous^35^ (image quality in Figure 2e was compromised by thermal drift, but is restored in a subsequent MFM image, Supporting Information Note 4). A high-temperature excursion to the cubic C-phase of BTO is uninteresting because it irreversibly erases stripe domains (Supporting Information Note 5).

To investigate the electrically driven magnetic changes microscopically, we used PEEM with XMCD contrast to image the Ni film near a zig-zag edge (see Experimental Section), and we used PEEM with X-ray linear dichroism (XLD) contrast^36^ to image nearby ferroelectric domains in the exposed BTO substrate (**Figure 3**). The definition of an edge was necessary because ferroelectric domains cannot be imaged through our 100-nm-thick films using either PEEM, birefringence, or piezoforce microscopy (PFM); and because thinner films show no stripe domains (Supporting Information Note 6).

**Figure 3:**
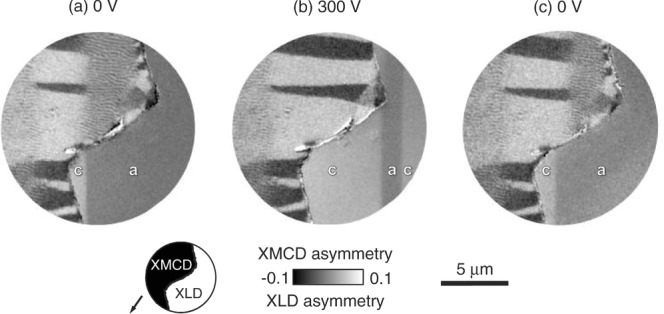
Concomitant electrical control of stripe domains and ferroelectric domains. Composite images obtained at room temperature for a) 0 V, b) 300 V, and c) 0 V following an initial electrical cycle (Supporting Information Note 7). As shown in the schematic, these images were spliced together on either side of a zig-zag edge in the film, thus combining a PEEM image of the film obtained with XMCD contrast, and a PEEM image of the exposed substrate obtained with XLD contrast. Inferred and observed *a* and *c* domains of BTO are labeled. Arrow shows IP projection of incident-beam direction. Data for Sample 5.

For this magnetoelectric imaging, a room-temperature electrical cycle (Supporting Information Note 7) produced our starting configuration (Figure 3a) in which a ferroelectric domain wall meets the zig-zag edge of the film. The pale ferroelectric domain is associated—by extrapolation across the zig-zag edge—with ferromagnetic domains possessing IP magnetizations. (IP domains near the zig-zag edge arise due to relief of growth stress by delamination.) The observed ferroelectric domain wall, and the collinear annihilation front in the Ni film, undergo together an electrically driven displacement (Figure 3b) that is essentially reversible (Figure 3c) (Supporting Information Note 8 shows similar data for a second edge). The pale ferroelectric domain is *c*-oriented, as a pale domain dominated in ±300 V (Figure S8e,i, Supporting Information Note 7). Therefore we infer that the electrical annihilation of stripe domains in favor of IP domains is due to the conversion of underlying *a* to *c* domains.

A widespread MFM investigation of Samples 1–4 was sufficient to reveal many regions where stripe domains could be electrically switched off and on in a non-volatile manner (e.g., **Figure 4**). Unlike our thermally driven magnetic changes (Figure 2), these magnetoelectric effects are not ubiquitous because 90° switching in T-phase BTO is somewhat inhibited by stress from other domains.^34^ Therefore, macroscopic magnetoelectric effects (discussed in Supporting Information Note 9) are localized and small.

**Figure 4:**
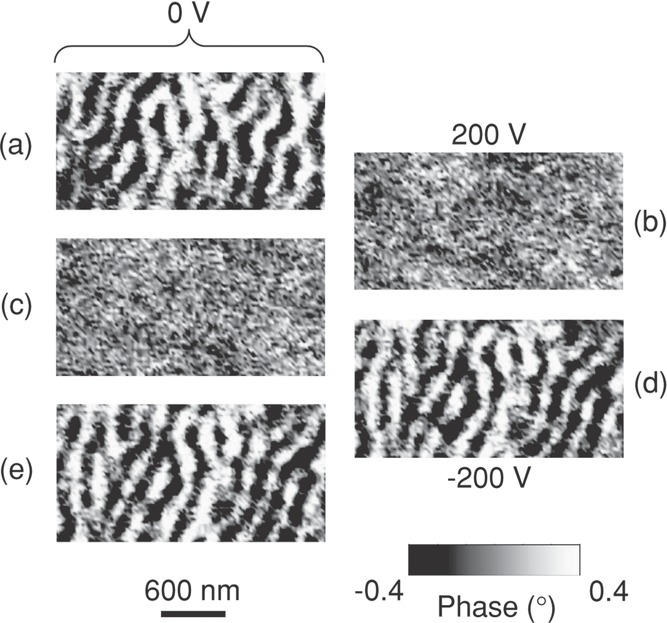
Repeatable and non-volatile electrical control of stripe domains. Flattened MFM images obtained at room temperature for a) 0 V, b) 200 V, c) 0 V, d) –200 V, and e) 0 V. Data for Sample 1.

We next use an essentially qualitative analysis, then a quantitative analysis, to show that thermally/electrically annihilated stripe domains lie on T-phase *a* domains. We will see that thermal/electrical annihilations both arise due to local IP strains that are nominally uniaxial, compressive, and similar in magnitude (≈1%). The resulting uniaxial stress eliminates the OOP anisotropy created by isotropic stress provided its magnitude is equal or larger (Supporting Information Note 10).

We first use a qualitative argument to deduce that thermally annihilated stripe domains lie on T-phase *a* domains. On cooling BTO from T to R, lattice parameter 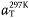 increases 0.1%, and lattice parameter 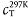 reduces 0.97%. For an 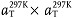 template presented to the film by T-phase *c* domains, cooling to 127 K produces an isotropic IP expansion that would enhance not annihilate the OOP stress anisotropy (with additional enhancement because substrate clamping generates tensile stress by preventing film thermal contraction). For the 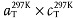 template of *a* domains, cooling to 127 K produces a nominally uniaxial ≈1% contraction, annihilating the OOP stress anisotropy (Supporting Information Note 10) (the opposing ≈0.17% thermal contraction^37^ from Ni may be ignored). Therefore stripe domains that undergo thermal annihilation lie on T-phase *a* domains whose twinning results in biaxial anisotropy (Supporting Information Note 2).

An essentially qualitative argument shows that electrically annihilated stripe domains also lie on T-phase *a* domains, as 90° switching interconverts *a* domains (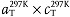) and *c* domains (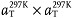), but only *a* → *c* yields the requisite interfacial contraction (≈1%).

We now present a quantitative analysis to explain our experimental observations of stripe-domain annihilation. The tensile isotropic IP growth stress 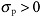 may be evaluated from uniaxial OOP anisotropy^29^ 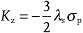 (saturation magnetostriction 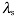 = −32.9 × 10^−6^ for room-temperature Ni). For our 125 nm-wide stripes, the micromagnetic theory of stripe domains implies 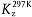 = 22 kJ m^−3^ (Supporting Information Note 11). Alternatively, variable-temperature IP and OOP measurements of macroscopic magnetization along virgin curves imply 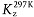 = 24 kJ m^−3^ (Supporting Information Note 12). These values of 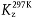 are similar, and in the 10–60 kJ m^−3^ range for Ni films.^26^,^27^ The corresponding value of 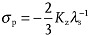 ≈0.5 GPa is large, but typical of the growth stress in polycrystalline metallic films,^31^ and similar to the value required to create stripe domains in a Ni-rich film.^38^ Even if the predicted ≈1% contractions are not fully developed in the twinned substrate or fully transmitted to the film, this 0.5 GPa growth stress is indeed small enough to be overcome by our electrically/thermally generated stresses, as the electrically driven ≈1% strain corresponds to a stress of 1.3 GPa, and the thermally driven ≈1% strain corresponds to an even larger stress as the 133 GPa Young's modulus of Ni increases on cooling. (Reversible piezoelectric strain at 200 V is two orders of magnitude smaller and may be ignored, Supporting Information Note 13.)

In summary, we have demonstrated systematic electrical and thermal control of OOP magnetization, using growth stress to create the requisite OOP anisotropy, and using stress associated with discontinuous changes of lattice parameter to annihilate this anisotropy. Our magnetoelectric imaging is attractive as images of both ferromagnetic and ferroelectric domains are rare;^13^,^18^ concomitant changes have only been seen in low-resolution studies;^13^ and published PEEM data have hitherto exploited XMCD contrast without XLD contrast^17^,^18^,^39^ except for one single image.^36^ Our high-resolution magnetoelectric measurements are suitable for nanostructures that are too small for magneto-optical Kerr effect and birefringence.^13^ Moreover, our approach is preferable to scanning force microscopy with both^24^ MFM and PFM, because the magnetic information is more direct and complete, and because the two imaging modes may be trivially swapped at any temperature.

In future, one may use nanopatterned bilayers on inactive substrates for non-volatile voltage control of magnetization at a precise array of locations. If this were achieved by exploiting films patterned down to, for example, the stripe width, then one may electrically switch OOP magnetization in bits that each comprise a single magnetic domain, notably for low-power write in high-density perpendicular data storage.

## Experimental Section

*Sample Preparation and Structural Charaterization*: All six samples comprised 100-nm-thick Ni films grown on 4 mm × 4 mm × 0.5 mm single-crystal BTO (001) substrates using room-temperature e-beam assisted evaporation with a base pressure of 1.5 × 10^−10^ mbar. All films were capped with 4 nm of Cu to prevent oxidation. The Ni deposition rate was ≈0.3 nm min^−1^, as determined using a quartz microbalance. Films were either co-deposited (Samples 1 and 2; Samples 5 and 6) or deposited separately (Samples 3 and 4). For Samples 1–4, we used unpoled BTO substrates. For Samples 5 and 6, which were prepared for both the initial decoration study and the magnetoelectric imaging, we poled substrates prior to growth by applying 300 V (6 kV cm^−1^) between a sputter-deposited back electrode of Pt and a sacrificial top electrode of 8-nm-thick evaporated Au. The poling process was monitored through this layer of Au by optical birefringence. After etching away the Au, small areas of the BTO surface were protected during Ni deposition using a marker pen. Lift-off in isopropyl alcohol subsequently exposed these small areas, which were bounded by film edges that were sometimes zig-zag. Using a Philips powder diffractometer with Cu K_α_ radiation to study Sample 1, we found a lateral Ni grain size of over 100 nm, and no evidence for preferred orientations.

*Magnetic Measurements*: Magnetization measurements were performed using a Princeton Measurements Corporation vibrating sample magnetometer (VSM), with electrical access to the sample.^40^ Measurements of macroscopic IP magnetization were performed after initially rotating the sample to maximize remanence.

*Magnetic Force Microscopy*: MFM measurements were performed in a Digital Instruments Dimension 3100, at lift heights of 40–60 nm, using low-moment ASYMFMLM Asylum Research tips of stiffness 2 N m^−1^ coated with 15 nm of CoCr. All MFM images were obtained at magnetic remanence after having applied an OOP magnetic field, except for Figure 4b, Supporting Information Note 3, which was obtained while an IP magnetic field was applied using a tapered soft magnet connected to a larger permanent magnet. All MFM image analysis was performed using WSxM software.^41^

*Photoemission Electron Microscopy*: PEEM in zero magnetic field was performed with the X-ray beam at a grazing-incidence angle of 16°, using an Elmitec SPELEEM-III microscope on beamline I06 at Diamond Light Source. The probe depth was ≈7 nm, and the lateral resolution was typically ≈50 nm. Images of ferromagnetic (ferroelectric) domains without topographical information were obtained by plotting average values of XMCD (XLD) asymmetry. These values represent the projection onto the incident-beam direction of the local surface magnetization (polarization), whose IP and OOP components are both detected. For images acquired with right (R) and left (L) circularly polarized light, the XMCD asymmetry of each pixel is given by 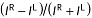, where 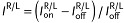 is the relative intensity for secondary-electron emission arising from X-ray absorption on (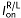 at 851 eV) and off (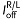 at 842 eV) the Ni *L*_3_ resonance. For images acquired with vertically (V) and horizontally (H) polarized light, the XLD asymmetry of each pixel is given by 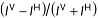, where 
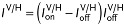
 is the relative intensity for secondary-electron emission arising from X-ray absorption on (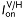 at 457 eV) and off (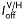 at 446 eV) the Ti *L*_3_ resonance. The comparison between intensities obtained on and off resonance avoids the influence of any inhomogeneous illumination. Images for each X-ray energy and beam polarization were acquired during 5 s exposure times. Each XMCD–PEEM image that we present was constructed via an averaging process based on 20 such images, but XMCD–PEEM and XLD–PEEM images that we present together were each constructed via an averaging process based on 40 such images.

Voltage *V* was applied during VSM, PEEM, and MFM measurements between the grounded Ni film, and a sputter-deposited Pt electrode under the BTO substrate.
