# Supplementary material for: Perpendicular Local Magnetization Under Voltage Control in Ni Films on Ferroelectric BaTiO3 Substrates
Source: Adv Mater. 2015 Jan 12;27(8):1460–5. doi: 10.1002/adma.201404799 (PMC4515098; doi:10.1002/adma.201404799)
Supplement: Supplementary file 1 [file adma0027-1460-sd1.pdf]

Copyright WILEY-VCH Verlag GmbH & Co. KGaA, 69469 Weinheim, Germany, 2015.

ADVANCED MATERIALS

Supporting Information

for *Adv. Mater.,* DOI: 10.1002/adma.201404799

Perpendicular Local Magnetization Under Voltage Control in Ni Films on Ferroelectric $BaTiO_3$ Substrates

*Massimo Ghidini, Francesco Maccherozzi, Xavier Moya, Lee C. Phillips, Wenjing Yan, Jordane Soussi, Nicolas Métallier, Mary E. Vickers, Nina -J. Steinke, Rhodri Mansell, Crispin H. W. Barnes, Sarnjeet S. Dhesi, and Neil D. Mathur**

Supporting Information

Perpendicular local magnetization under voltage control

in Ni films on ferroelectric $BaTiO_3$ substrates

*Massimo Ghidini, Francesco Maccherozzi, Xavier Moya, Lee C. Phillips, Wenjing Yan, Jordane Soussi, Nicolas Métallier, Mary E. Vickers, Nina -J. Steinke, Rhodri Mansell, Crispin H. W. Barnes, Sarnjeet S. Dhesi, Neil D. Mathur**

Contents

Page	Supplementary Note	
3	1	Stripe domains in 100 nm-thick Ni films on various substrates
4	2	Globally biaxial IP magnetic anisotropy at 127 K
5	3	Long thin features with IP magnetization decorate *c* domains in Sample 1
7	4	Recovery of stripe domains after the excursion to 127 K
8	5	Irreversible erasure of stripe domains after exceeding the BTO Curie temperature
9	6	Influence of film thickness on the formation of stripe domains and stripe width
10	7	Initial electrical cycle showing non-local annihilation of stripe domains
12	8	Decoration study near a second edge
13	9	Macroscopic magnetoelectric effects
14	10	Elimination of stripe domains due to uniaxial IP compressive stress
18	11	OOP anisotropy constant from microscopic data
19	12	OOP anisotropy constant from macroscopic data
20	13	Reversible piezoelectric strain in BTO
21		References S1-S8

Supplementary Note 1

Stripe domains in 100 nm-thick Ni films on various substrates

Magnetic stripe domains have previously been seen in 100 nm-thick films of Ni on glass[26] and silicon.[27] Here we report magnetic stripe domains in 100 nm-thick films of Ni grown as described in Methods on silicon, quartz and 0.72Pb($Mg_{1/3}Nb_{2/3}$)O_3–0.28$PbTiO_3$ (PMN-PT). We see that substrate choice does not influence the presence of stripe domains or stripe width.

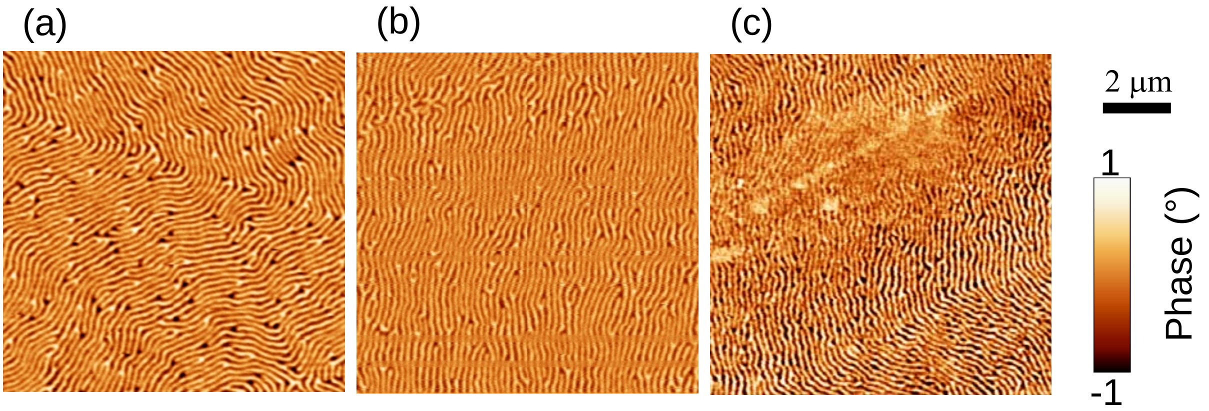

Fig. S1. Stripe domains in Ni films on various substrates. MFM images (10 µm × 10 µm) of 100 nm-thick films on (a) silicon, (b) quartz, and (c) PMN-PT.

Supplementary Note 2

Globally biaxial IP magnetic anisotropy at 127 K

At room temperature, there is no significant IP magnetic anisotropy, and loop squareness is correspondingly low (M_r/M_s ~ 0.4, M_r is remanent magnetization, M_s is saturation magnetization). On cooling to 127 K, the two species of *a* domains that dominate our substrates undergo nominally uniaxial contractions along their mutually perpendicular IP *c* axes. Therefore the resulting IP anisotropy is globally biaxial, and loop squareness is correspondingly increased at all measurement angles.

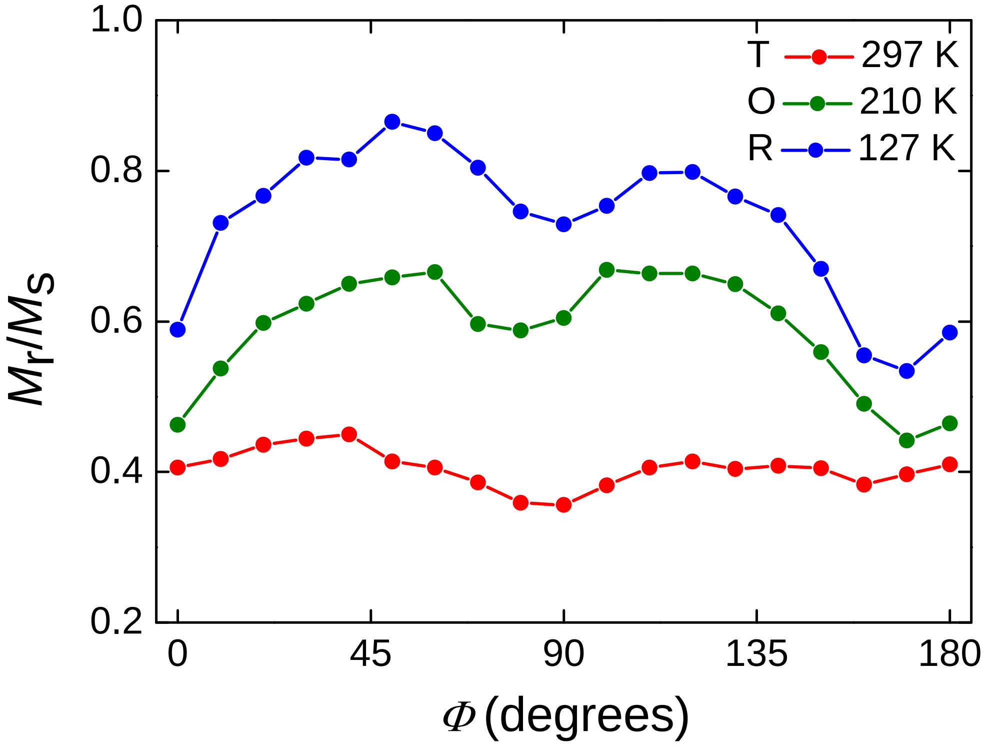

Fig. S2. Changes in magnetic anisotropy on cooling. The squareness M_r/M_s of magnetic hysteresis loops obtained for various IP measurement directions Φ is plotted for 297 K (red), 127 K (blue) and 210 K (green). The hysteresis loops obtained at Φ ~ 45° are shown in Fig. 1b. Data for Sample 1.

Supplementary Note 3

Long thin features with IP magnetization decorate *c* domains in Sample 1

For Sample 1 alone, most of many MFM images reveal a long thin feature with an IP magnetization (e.g. Fig. 2a). Each such feature decorates a ferroelectric domain in the BTO substrate, as its sides are straight (Fig. 2a, S3a, S4a), it is modified by an electric field (that reduces feature width and generates stripe domains, Fig. S3), and it is not modified by a magnetic field (that merely aligns the average stripe orientation due to rotatable anisotropy,[26] Fig. S4).

This decoration of ferroelectric domains is reminiscent of as-grown polycrystalline $Co_{60}Fe_{40}$ films whose IP magnetization decorates BTO twins,[13] and is surprising as stripe domains should be ubiquitous in our as-grown samples even though they are twinned, given that substrate choice does not influence the presence of stripe domains or stripe width (Fig. S1). The decoration could arise if the growth stress responsible for stripe domains were modified in the early stages of deposition by an electric field emanating from *c* domains with OOP polarization, cf. the use of electric fields to modify the growth of thin metallic films.[S1] However, the significant number of *c* domains in Samples 5-6 were not decorated by an IP magnetization, which may be due to the effect on the BTO surface of the sacrificial Au layer that was temporarily present for pre-poling.

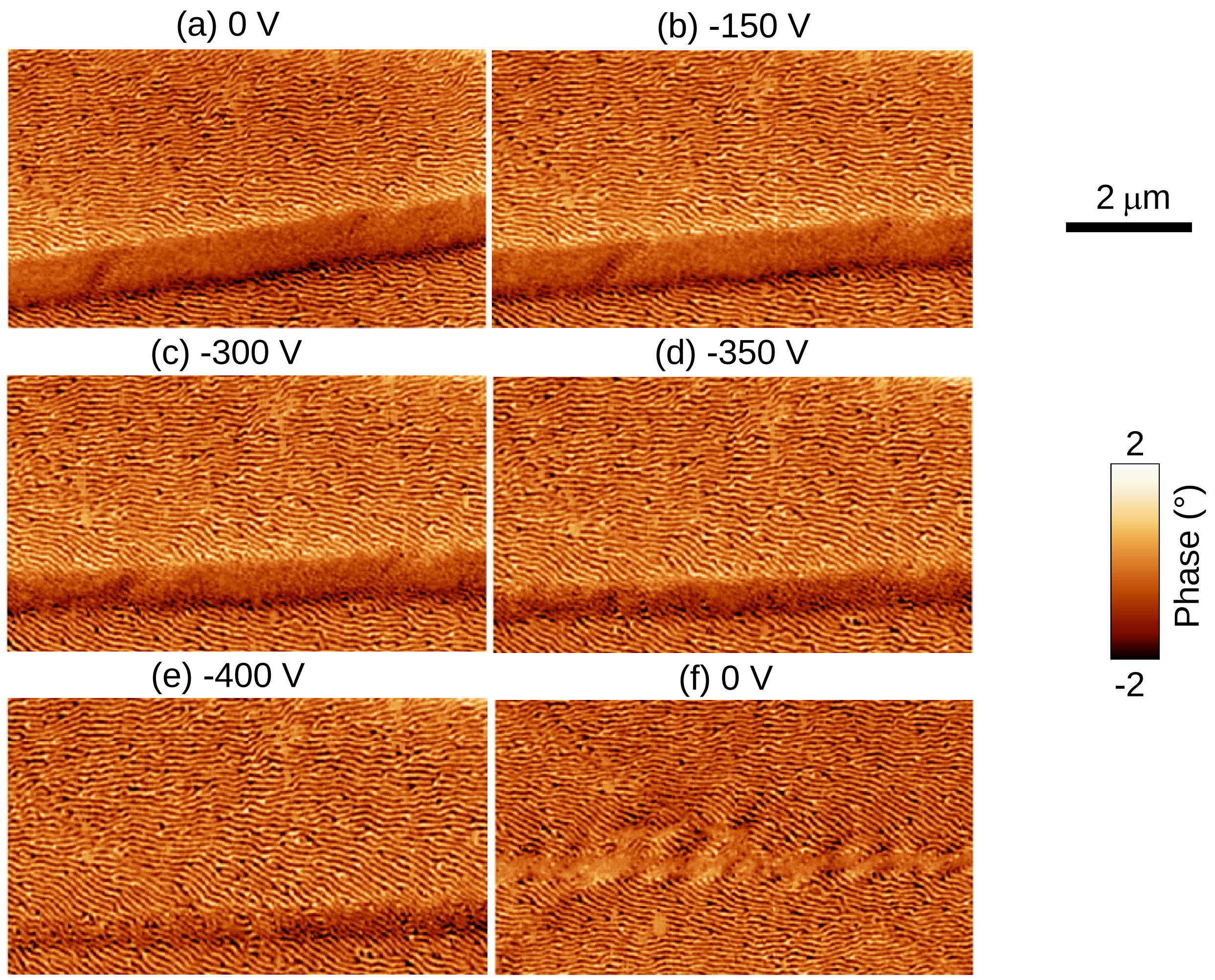

Fig. S3. Influence of electric field on a long thin feature in Sample 1. Flattened MFM images (20 μm × 11 μm) obtained at room temperature while cycling the applied voltage from 0 to -400 V to 0 V.

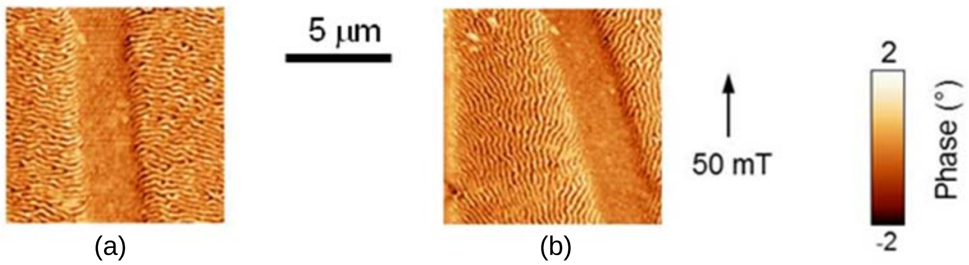

Fig. S4. No influence of magnetic field on a long thin feature in Sample 1. Flattened MFM images (10 μm × 10 μm) obtained at room temperature (a) before and (b) during the application of 50 mT along the arrowed IP direction. A sample drift of ~2 μm is apparent. The stripe orientation in (b) was essentially unchanged on removing the magnetic field (Fig. S3a).

Supplementary Note 4

Recovery of stripe domains after the excursion to 127 K

When stripe domains are restored after the thermal cycle of Fig. 2, the stripe width is recovered and the waviness is reduced.

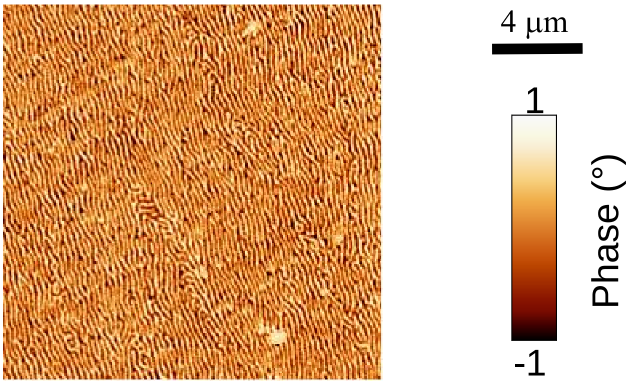

Fig. S5. Stripe domains at room temperature after a low-temperature thermal excursion. Flattened MFM image (20 μm × 20 μm) of approximately the same region seen in Fig. 2 of the main paper.

Supplementary Note 5

Irreversible erasure of stripe domains after exceeding the BTO Curie temperature

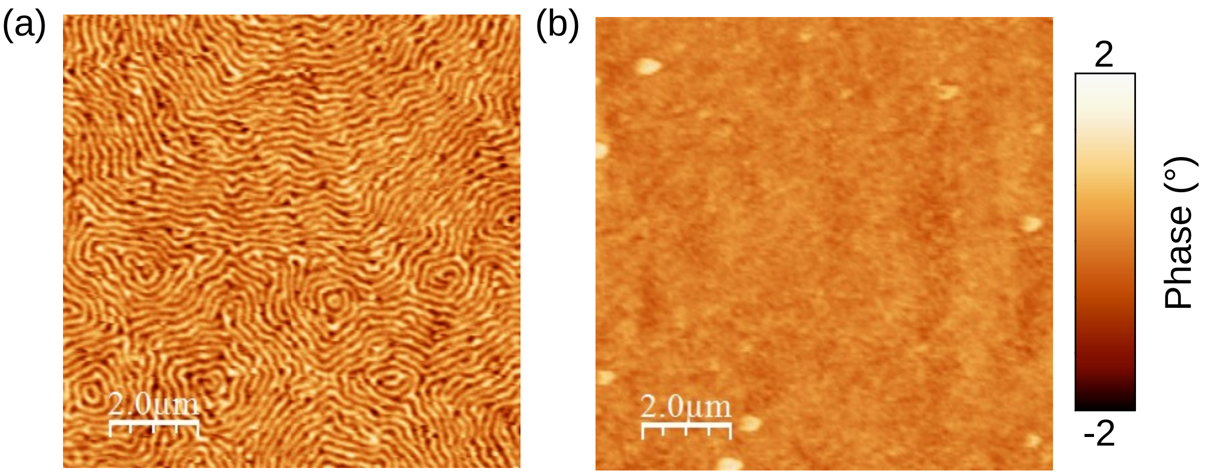

Fig. S6. Irreversible erasure of stripe domains. Flattened MFM images (10 µm × 10 µm) obtained at room temperature for (a) the as-deposited state, and (b) after an excursion above the BTO Curie temperature of ~400 K. Data for Sample 3.

Supplementary Note 6

Influence of film thickness on the formation of stripe domains and stripe width

Ni films of various thicknesses were grown on silicon substrates as described in Methods, but different growth rates were employed. As expected,[27] magnetic stripe domains appear above a critical film thickness (100 nm), and stripe width is approximately equal to film thickness[29] and independent of growth rate.

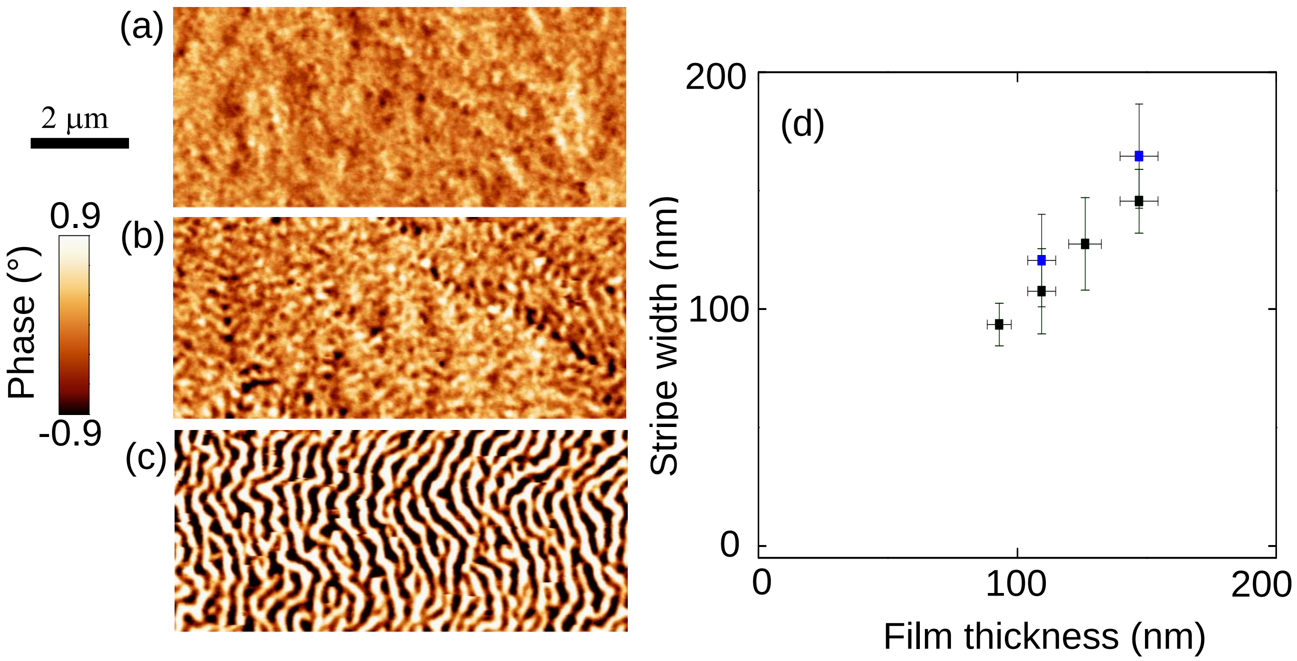

Fig. S7. Influence of film thickness on stripe domains. Flattened MFM images (9 μm × 4 μm) obtained at room temperature for Ni films of thickness (a) 84 nm, (b) 109 nm and (c) 147 nm, grown on silicon substrates at 4.6 nm s^{-1}. (d) Stripe width versus Ni thickness for the films represented in (b,c) (blue squares), and for films of thickness 96 nm, 109 nm, 126 nm and 147 nm grown on silicon substrates at 0.46 nm s^{-1} (black squares).

Supplementary Note 7

Initial electrical cycle showing non-local annihilation of stripe domains

Prior to collecting the data of Fig. 3 in a 15 μm field of view, we collected data during an initial electrical cycle in a 50 μm field of view that was centred at the same location (Fig. S8). The unresolved stripe domains in the larger field of view appear grainy.

The initial application of 100 V (Fig. S8a→b) results in the non-volatile annihilation of stripe domains in a region that lies on a *c* domain rather than an *a* domain. We attribute this annihilation to in-plane compressive stress generated in the *c* domain by the formation of a nearby *a* domain in the presence of clamping from other domains. Given that the *a* domain imparts compressive stress to the *c* domain, we may infer that its long *c* axis lies horizontally in the field of view, and therefore perpendicular to the ferroelectric domain wall between *a* and *c* domains, as expected[13]. The subsequent annihilation of stripe domains was volatile and is associated with the conversion of *a* to *c* domains (Fig. S8b→j and then in Fig. 3), as discussed in the main paper.

The left-hand boundary of the region in which stripe domains were annihilated by the initial application of a voltage (Fig. S8b) is likely to coincide with an immobile ferroelectric domain wall, given the following two observations that we made when the stripe domains were present (Fig. S8a). First, it was possible to observe a change of image contrast across the boundary due to the corrugation associated with *a*-*c* twins (the change is weak because the 0.6° corrugation angle[S2] is small with respect to the grazing-incidence angle of 16°, and because the IP projection of the beam is not perpendicular to the ferroelectric domain wall). Second, there was a topographical discontinuity in the PEEM image that corresponds to Fig. S8a when contrast was provided by x-ray absorbtion spectroscopy (XAS) rather than XMCD.

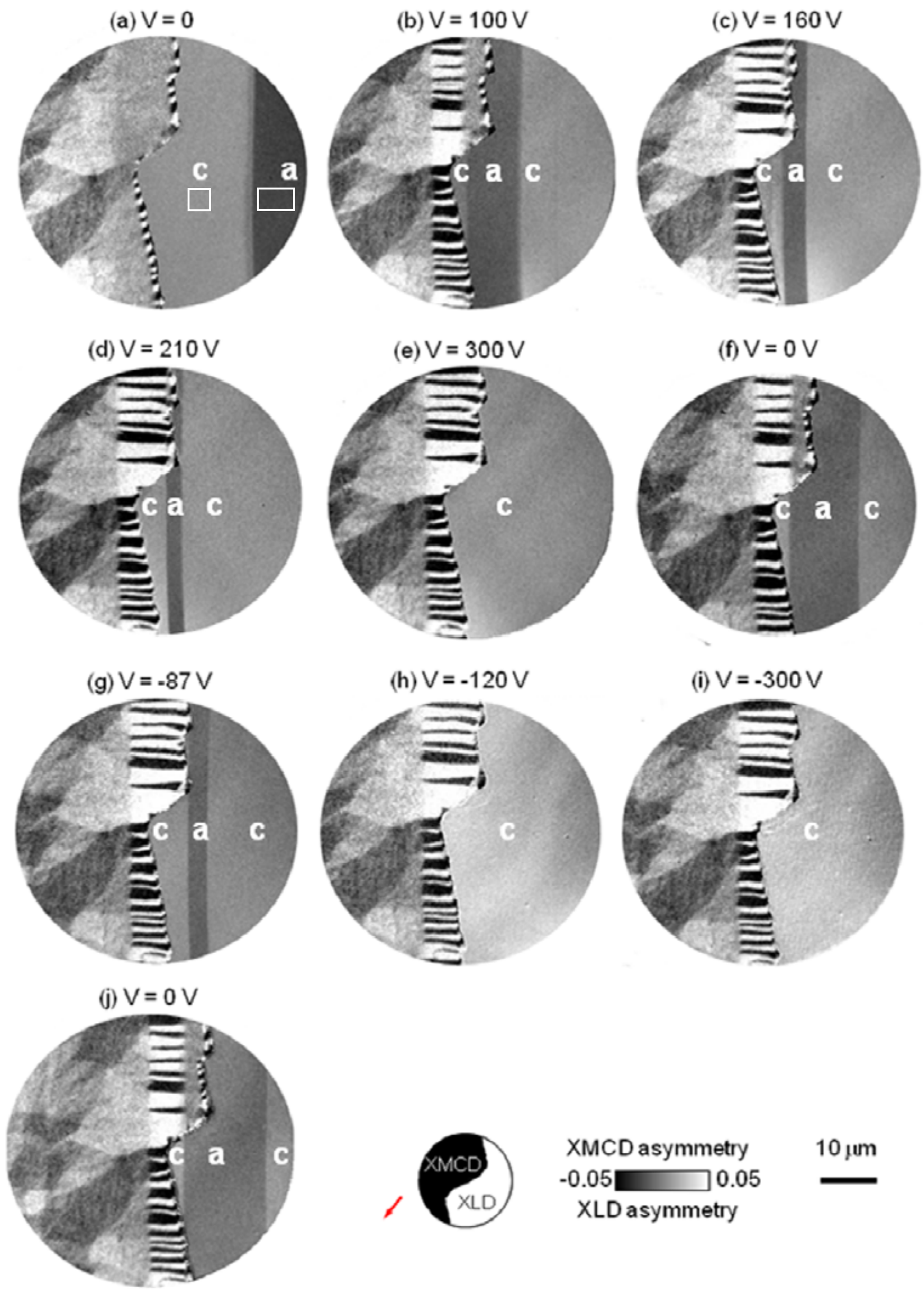

Fig. S8. **Initial electrical cycle obtained prior to Fig. 3.** Composite images obtained at room temperature for the voltages indicated, using a 50 μm field of view that was centred as for Fig. 3 [(sample drift is apparent in (j)]. As shown in the schematic, these images were spliced together on either side of a zig-zag edge in the film, thus combining a PEEM image of the film obtained with XMCD contrast, and a PEEM image of the exposed substrate obtained with XLD contrast. Exposed *a* and *c* domains of BTO are labelled. In (a), the shape of the BTO unit cell is indicated using a rectangle for *a* domains, and a square for *c* domains. Red arrow shows IP projection of incident-beam direction. Data for Sample 5.

Supplementary Note 8

Decoration study near a second edge

After obtaining the data of Fig. 3, we obtained similar data from the same sample, using a slightly larger field of view to study a second film edge (Fig. S9). The voltage annihilates stripe domains and the *a* domain with which they are associated, in favour of IP magnetic domains associated with what is now a single *c* domain (Fig. S9b). After removing the voltage, the region that originally contained stripe domains, and the *a* domain with which they are associated, is expanded (Fig. S9c). The original stripe domains in the film have therefore undergone an electrically driven annihilation that is reversible.

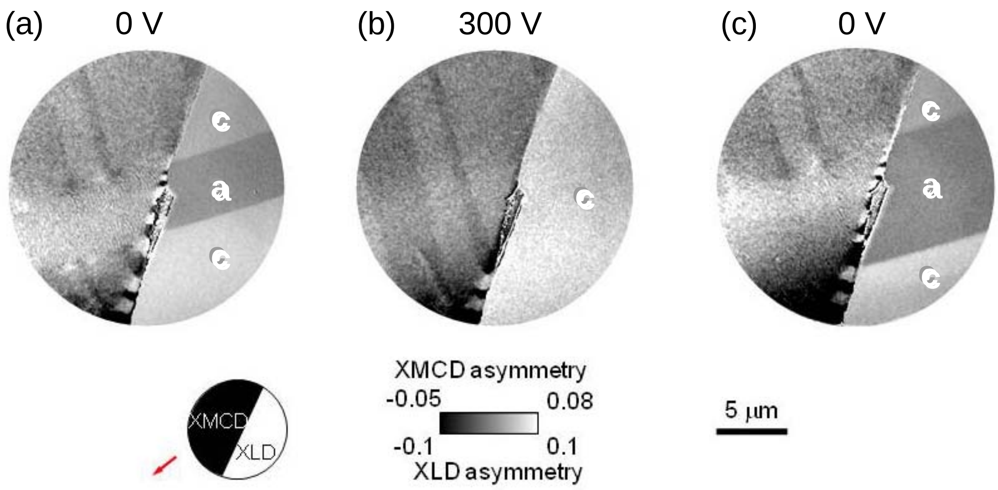

Fig. S9. **Concomitant electrical control of stripe domains and ferroelectric domains near a second edge.** Composite images obtained at room temperature for (a) 0 V, (b) 300 V, and (c) 0 V, at a new location after collecting the data of Fig. 3. As shown in the schematic, these images were spliced together on either side of a zig-zag edge in the film, thus combining a PEEM image of the film obtained with XMCD contrast, and a PEEM image of the exposed substrate obtained with XLD contrast. Exposed *a* and *c* domains of BTO are labelled. Red arrow shows IP projection of incident-beam direction. Data for Sample 5.

Supplementary Note 9

Macroscopic magnetoelectric effects

The application of 300 V (6 kV cm^{-1}) exceeds the coercive voltage of a typical BTO substrate (Fig. S10). This voltage leads to small increases of easy-axis loop squareness (Fig. S11a) consistent with stripe-domain annihilation when canted moments are electrically rotated to lie in plane. This voltage also leads to small increases of hard-axis saturation field (Fig. S11b) consistent with the reduction of OOP anisotropy that results in stripe-domain annihilation (the voltage does not modify the remanent magnetization as a set of stripe domains possesses no net OOP magnetization). The complex nature of ferroelectric domain swithing in BTO results in a small electrical hysteresis that is apparent in Fig. S11a,b.

Fig. S10. Ferroelectric hysteresis in a BTO substrate. Polarisation *P* versus applied electric field *E* measured via the constant-current method[S3] using a Keithley 2400 SourceMeter. Applied voltage *V* is also shown.

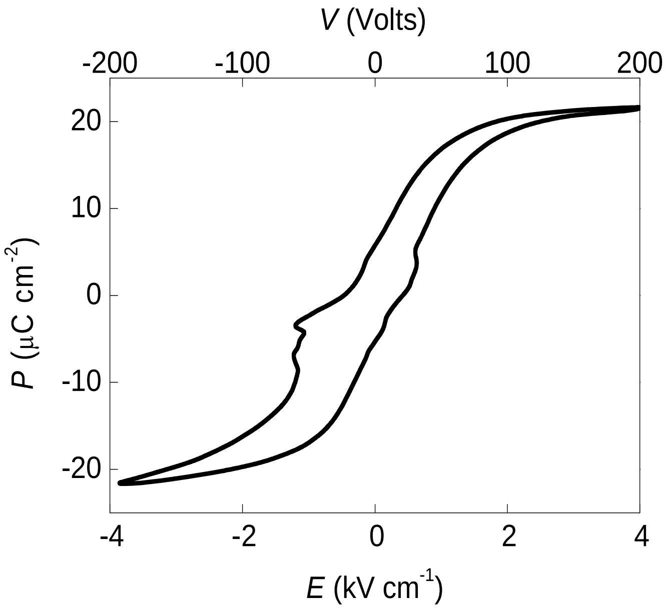

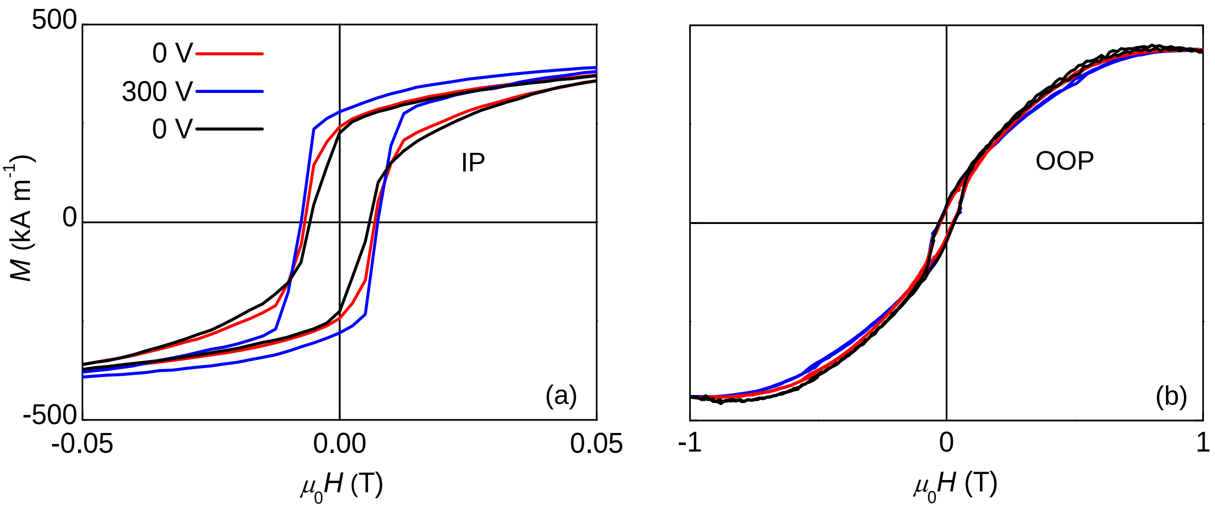

Fig. S11. Small macroscopic magnetoelectric effects. Magnetization *M* versus applied magnetic field *H*, for (a) IP and (b) OOP directions, at 0 V (red), 300 V (blue), and then 0 V (black). Data for Sample 2.

Supplementary Note 10

Elimination of stripe domains due to uniaxial IP compressive stress

Our stripe domains arise in negative-magnetostriction Ni ($\lambda_s < 0$) because of the OOP anisotropy that results from *isotropic IP* tensile growth stress $\sigma_p > 0$. Here we show that *uniaxial IP* compressive stress $\sigma_u < 0$ eliminates the OOP anisotropy required for stripe domains if $|\sigma_u| \geq |\sigma_p|$. We demonstrate this by considering the magnetoelastic energy of a polycrystalline ferromagnet:[S4]

$$E_{me} = -\frac{3}{2}\lambda_s \sum_{i,k} \sigma_{ik}\left(m_i m_k - \frac{1}{3}\delta_{ik} \right),$$

where m_i are the direction cosines of **M**. In what follows, λ_s will be replaced by $-|\lambda_s|$ as $\lambda_s < 0$; principal axes i = 1, 2, 3 are denoted x, y, z respectively; and the growth stress lies in the x-y plane with the OOP direction along $+z$.

Isotropic IP tensile growth stress

We have $\sigma_{xx} = \sigma_{yy} = \sigma_p$, so E_{me} is given by:

$$E_{iso} = \frac{3}{2}|\lambda_s|\left(\sigma_{xx}\left(m_x^2 - \frac{1}{3} \right) + \sigma_{yy}\left(m_y^2 - \frac{1}{3} \right) \right) = \frac{3}{2}|\lambda_s|\sigma_p\left(\frac{1}{3} - m_z^2 \right).$$

However, the absolute value is irrelevant so we will neglect the constant term and instead write:

$$\boxed{E_{iso} = -\frac{3}{2}|\lambda_s|\sigma_p m_z^2}.$$

Given that $\sigma_p > 0$, this favours a perpendicular easy-axis ($m_z = \pm 1$).

Uniaxial IP compressive stress along x

We have $\sigma_{xx} = \sigma_u$, so E_{me} is given by:

$$\boxed{E_u = \frac{3}{2}|\lambda_s|\sigma_u m_x^2}.$$

Given that $\sigma_u < 0$, this favours an IP magnetization along x ($m_x = \pm 1$).

Stable directions of **M** for both stresses together

The total magnetoelastic energy is given by:

$$E_{\text{iso}} + E_{\text{u}} = \frac{3}{2}|\lambda_{\text{s}}|\left(\sigma_{\text{u}} m_x^2 - \sigma_{\text{p}} m_z^2\right) = \frac{3}{2}|\lambda_s|\sigma_p U ,$$

where: $\boxed{U = k m_x^2 - m_z^2}$,

with $k = \frac{\sigma_{\text{u}}}{\sigma_{\text{p}}}$, where $\sigma_{\text{p}} > 0$ and $\sigma_{\text{u}} < 0$ such that $k < 0$. To find minima in $U(m_x, m_y, m_z)$, we will investigate stationary states by expressing U in terms of two of the three variables at a time (the three variables are interrelated via $m_x^2 + m_y^2 + m_z^2 = 1$).

a) $U(m_x, m_z)$

We have: $\frac{\partial U}{\partial m_x} = 2k m_x = 0$ $\frac{\partial U}{\partial m_z} = -2m_z = 0$

such that: $m_x = 0$ $m_z = 0$

Therefore $m_y = \pm 1$ such that **M** lies along $\pm y$. However, the diagonal Hessian matrix describing this equilibrium has eigenvalues $2k$ and -2:

$$\begin{bmatrix} \frac{\partial^2 U}{\partial m_x{}^2} & \frac{\partial^2 U}{\partial m_z \partial m_x} \\ \frac{\partial^2 U}{\partial m_z \partial m_x} & \frac{\partial^2 U}{\partial m_z{}^2} \end{bmatrix} = \begin{bmatrix} 2k & 0 \\ 0 & -2 \end{bmatrix}$$

Given that both eigenvalues are negative, **M** along $\pm y$ represents *unstable* equilibrium.

b) $U(m_x, m_y)$

From: $U = k m_x{}^2 - 1 + m_x{}^2 + m_y{}^2 = (k+1) m_x{}^2 + m_y{}^2 - 1$

we have: $\frac{\partial U}{\partial m_x} = 2(k+1) m_x = 0$ $\frac{\partial U}{\partial m_y} = 2m_y = 0$

such that: $m_x = 0$ $m_y = 0$

Therefore $m_z = \pm 1$ such that **M** lies along $\pm z$. The diagonal Hessian matrix describing this equilibrium has eigenvalues $2(k+1)$ and 2:

$$\begin{bmatrix} \frac{\partial^2 U}{\partial m_x^{\ 2}} & \frac{\partial^2 U}{\partial m_y \partial m_x} \\ \frac{\partial^2 U}{\partial m_y \partial m_x} & \frac{\partial^2 U}{\partial m_y^{\ 2}} \end{bmatrix} = \begin{bmatrix} 2(k+1) & 0 \\ 0 & 2 \end{bmatrix}$$

Both eigenvalues are positive if our negative value of $k = \frac{\sigma_{\mathrm{u}}}{\sigma_{\mathrm{p}}}$ lies in the range -1 < k < 0. Therefore **M** lies along $\pm z$ provided that $|\sigma_{\mathrm{u}}| < |\sigma_{\mathrm{p}}|$, i.e. provided that the magnitude of the uniaxial IP compressive stress is less than the magnitude of the isotropic IP tensile growth stress.

c) $U(m_y, m_z)$

From: $$U = 1 - km_y^{\ 2} - km_z^{\ 2} - m_z^{\ 2} = 1 - km_y^{\ 2} - (k+1)m_z^{\ 2}$$

we have: $$\frac{\partial U}{\partial m_y} = -2km_y = 0 \qquad \frac{\partial U}{\partial m_z} = -2(k+1)m_z = 0$$

such that: $$m_y = 0 \qquad m_z = 0$$

Therefore $m_x = \pm 1$ such that **M** lies along $\pm x$. The diagonal Hessian matrix describing this equilibrium has eigenvalues $-2k$ and $-2(k+1)$:

$$\begin{bmatrix} \frac{\partial^2 U}{\partial m_y^{\ 2}} & \frac{\partial^2 U}{\partial m_z \partial m_y} \\ \frac{\partial^2 U}{\partial m_z \partial m_y} & \frac{\partial^2 U}{\partial m_z^{\ 2}} \end{bmatrix} = \begin{bmatrix} -2k & 0 \\ 0 & -2(k+1) \end{bmatrix}$$

Both eigenvalues are positive if our negative value of $k = \frac{\sigma_{\mathrm{u}}}{\sigma_{\mathrm{p}}}$ exceeds unity in magnitude ($k < -1$). Therefore **M** lies along $\pm x$ provided that $|\sigma_{\mathrm{u}}| > |\sigma_{\mathrm{p}}|$, i.e. provided that the magnitude of the uniaxial IP compressive stress exceeds the magnitude of the isotropic IP tensile growth stress.

P.T.O.

Summary

We have investigated the behaviour of the magnetoelastic energy that results from combining isotropic IP tensile growth stress $\sigma_p > 0$ with uniaxial IP compressive stress $\sigma_u < 0$.

If $|\sigma_u| < |\sigma_p|$ then the magnetoelastic energy is minimized when **M** is collinear with the up/down OOP direction, demonstrating that the OOP stress anisotropy required for stripe domains remains present.

If $|\sigma_u| > |\sigma_p|$ then the magnetoelastic energy is minimized when **M** is collinear with the uniaxial IP stress, demonstrating that the OOP stress anisotropy required for stripe domains has been eliminated.

If $|\sigma_u| = |\sigma_p|$ then **M** is not stable along the up/down OOP direction, demonstrating that the OOP stress anisotropy required for stripe domains has been eliminated.

Therefore stripe domains are eliminated if $|\sigma_u| \geq |\sigma_p|$.

Supplementary Note 11

OOP anisotropy constant from microscopic data

For stripes of width W = 125 nm, in our films of thickness D = 100 nm with exchange stiffness[S5] $A = 0.82\times10^{-11}$ J m^{-1}, the room-temperature OOP anisotropy $\boxed{K_z^{297\,\mathrm{K}} = 22\ \mathrm{kJ\ m^{-3}}}$ was obtained from:

$$K_z^{297\,\mathrm{K}} = \frac{\pi^2}{D^2}\left(\frac{D^2}{W^2}+1\right)^2 A\,.$$

This expression was established by eliminating h from the following two expressions: [S6]

$$\frac{D}{W_\mathrm{b}} = \frac{1}{1-h} \qquad \text{and} \qquad \frac{D}{W} = \sqrt{\frac{1+h}{1-h}}\ ,$$

which are valid for a small quality factor $q = K_z^{297\,\mathrm{K}} / K_{xy}^{297\,\mathrm{K}} \sim 0.2$. Here, $W_\mathrm{b} = 2\pi\sqrt{A/K_z^{297\,\mathrm{K}}}$ is Bloch-wall width, $h = H_\mathrm{c} / \left(2K_z^{297\,\mathrm{K}} / \mu_0 M_\mathrm{s}\right)$ is the reduced critical field, H_c is the critical field for stripe-domain nucleation, $K_{xy}^{297\,\mathrm{K}} = \frac{1}{2}\mu_0\left(M_\mathrm{s}^{297\,\mathrm{K}}\right)^2$ is the easy-plane shape anisotropy that competes with $K_z^{297\,\mathrm{K}}$, μ_0 is the permeability of free space, and $M_\mathrm{s}^{297\,\mathrm{K}}$ = 434.5 kA m^{-1} (Fig. S12a, P.T.O.) is the room-temperature saturation magnetization.

Supplementary Note 12

OOP anisotropy constant from macroscopic data

At any given temperature, the area between IP and OOP virgin magnetization curves yields the effective anisotropy[S7] $K_{\mathrm{eff}} = K_{xy} - K_z$ for homogeneously magnetized films, where K_z is the uniaxial OOP anisotropy that competes with easy-plane shape anisotropy $K_{xy} = \frac{1}{2}\mu_0 M_{\mathrm{s}}^2$. Assuming that the inhomogeneity due to the stripe domains introduces an offset into this equation, and assuming that this offset does not change between 297 K and 127 K given that there is only a small change in M_{s}, we obtain $\boxed{K_z^{297\,\mathrm{K}} = 24\ \mathrm{kJ\ m^{-3}}}$ from $K_{\mathrm{eff}}^{127\,\mathrm{K}} - K_{\mathrm{eff}}^{297\,\mathrm{K}} = \left(K_{xy}^{127\,\mathrm{K}} - K_z^{127\,\mathrm{K}}\right) - \left(K_{xy}^{297\,\mathrm{K}} - K_z^{297\,\mathrm{K}}\right)$, where $K_z^{127\,\mathrm{K}} = 0$ in the absence of stripe domains, $K_{\mathrm{eff}}^{297\,\mathrm{K}} = 62\ \mathrm{kJ\ m^{-3}}$ (Fig. S12a), $K_{\mathrm{eff}}^{127\,\mathrm{K}} = 94\ \mathrm{kJ\ m^{-3}}$ (Fig. S12b), $K_{xy}^{297\,\mathrm{K}} = 118\ \mathrm{kJ\ m^{-3}}$ using $M_{\mathrm{s}}^{297\,\mathrm{K}} = 434.5\ \mathrm{kA\ m^{-1}}$ (Fig. S12a), and $K_{xy}^{127\,\mathrm{K}} = 126\ \mathrm{kJ\ m^{-3}}$ using $M_{\mathrm{s}}^{127\,\mathrm{K}} = 449.0\ \mathrm{kA\ m^{-1}}$ (Fig. S12b)].

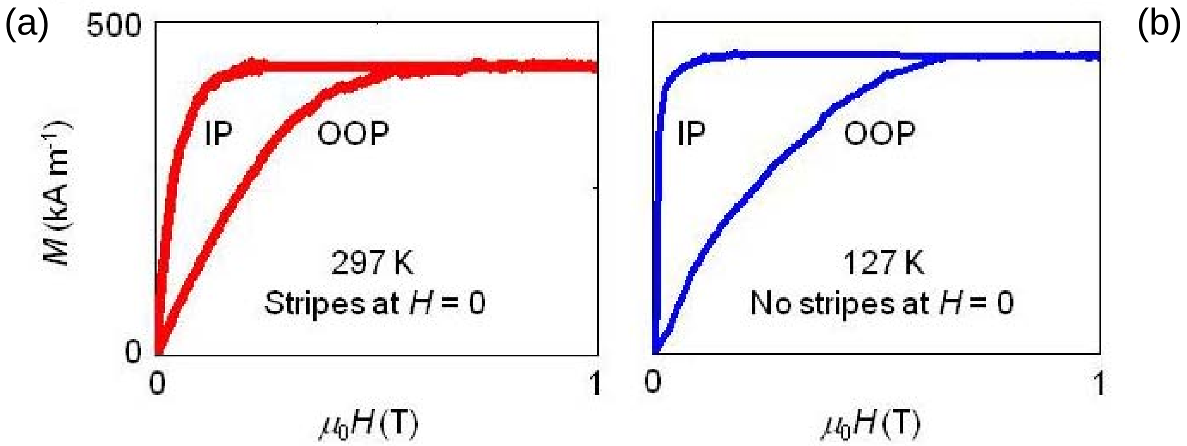

Fig. S12. Virgin magnetization measurements. IP and OOP magnetization M at (a) 297 K and (b) 127 K. This change of temperature eliminates stripe domains as seen in Fig. 2 of the main paper. Data for Sample 1 after demagnetization.

Supplementary Note 13

Reversible piezoelectric strain in BTO

The application of 200 V across our 0.5 mm-thick BTO substrates may generate reversible piezoelectric strains of $\varepsilon_1 = d_{31}E_3 = -3.2\times10^{-5}$ and $\varepsilon_3 = d_{33}E_3 = 5.8\times10^{-5}$, where[S8] $d_{33} = 149$ pm V^{-1} and $d_{31} = -82$ pm V^{-1}. These strains can be ignored as they are two orders of magnitude smaller than the ~1% uniaxial strain associated with the observed 90° ferroelectric domain switching.

Equally, the corresponding stresses of −4.3 MPa and 7.7 MPa are two orders of magnitude smaller than the $\sigma_p = 0.5$ GPa growth stress responsible for our stripe domains (the Young's modulus of Ni is 133 GPa at room temperature).

References S1-S8

[S1] K. L. Chopra, *J. Appl. Phys.* **1966**, *37*, 2249.

[S2] S. V. Kalinin, D. A. Bonnell, *J. Appl. Phys.* **2000**, *87*, 3950.

[S3] J. A. Giacometti, C. Wisniewski, W. A. Moura, P. A. Ribeiro, *Rev. Sci. Instrum.* **1999**, *70* 2702.

[S4] A. Hubert, R. Schäfer, page 138, *Magnetic Domains*, Springer-Verlag, Berlin, Germany, **1998**.

[S5] A. Michels, J. Weissmüller, A. Wiedenmann, J. G. Barker, *J. Appl. Phys.* **2000**, *87*, 5953.

[S6] A. Hubert, R. Schäfer, pages 299-306, *Magnetic Domains*, Springer-Verlag, Berlin, Germany, **1998**.

[S7] M. T. Johnson, P. J. H. Bloemenz, F. J. A. den Broeder, J. J. de Vries, *Rep. Prog. Phys.* **1996**, *59,* 1409.

[S8] R. Tazaki, D. Fu, M. Itoh, M. Daimon, Shin-ya Koshihara, *J. Phys.: Condens. Matter* **2009**, *21*, 215903.
